# Drinking water intake in feedlot cattle: a meta-analysis

**DOI:** 10.1093/jas/skag263

**Published:** 2026-09-01

**Authors:** Ian J Lean, Helen M Golder

**Affiliations:** Scibus, Camden, NSW 2570, Australia; School of Life and Environmental Sciences, Faculty of Science, The University of Sydney, Camden, NSW 2570, Australia; Scibus, Camden, NSW 2570, Australia; School of Life and Environmental Sciences, Faculty of Science, The University of Sydney, Camden, NSW 2570, Australia

**Keywords:** cattle, dry matter intake, feedlot, humidity, temperature, water intake

## Abstract

High-quality drinking water is critical for efficient feedlot production. We performed a systematic review and meta-analysis of drinking water intake (DWI) of cattle in feedlots and developed and evaluated models that predict DWI against industry standard predictions. Three search engines were used to identify experiments that included DWI in feedlot cattle. Results of experiments were evaluated to ensure that these were: from peer-reviewed journals or theses published in English; in vivo and evaluated use of DWI in feedlot cattle; had appropriate study designs; had appropriate analysis of data; and contained data to determine the effect size for outcomes. Environmental and production measures were extracted, and temperature humidity index (THI) and heat load index (HLI) were calculated. A mixed model meta-regression with the random effects of experiment within study was used to evaluate the univariate effects of temperature, THI, HLI, body weight, dry matter intake, average daily gain, dry matter percentage of the diet, sex, and shade on DWI. Multivariable, multilevel models to predict DMI were constructed by backwards stepping separately for temperature and THI when average daily temperature is >12°C. Existing equations for DWI for feedlot cattle were evaluated and compared to the models developed in this study by Lin’s concordance correlation coefficient ρ_c_, Pearson’s correlation, and Bland and Altman’s limits of agreement. Thirty-five studies with up to 131 experimental comparisons were included in the meta-analysis. The mean DWI was 31.2 L/d (SD = 4.31), mean temperature was 23.1°C (SD = 24.10), and temperature humidity index (THI) was 69.1 (SD = 9.62). Two models to predict DWI for feedlot cattle were developed, the first with temperature and body weight both independently predicting DWI (Root mean square error [RMSE] = 2.40 L/d) based on 20 studies and 75 experiments. The second model had THI and dry matter intake independently predicting DWI (RMSE = 2.83 L/d) based on 14 studies and 57 experiments. These models are consistent with estimates from other individual studies, but lower than those of the NRC. Drinking water intake decreased by 0.2 liter for every 100 mg/L increase in water sulfate (SO_4_). Shade reduced DWI by 3.4 L/d. We provide updated robust estimates of changes in DWI for feedlot cattle with increases in environmental temperature, THI, body weight, and feed intake to guide industry in water resource allocation.

## Teaser

Drinking water intake estimates obtained using 35 papers and 131 experiments show that feedlot cattle use less drinking water than currently used standards. Our estimates were based on studies involving a wide range of environmental conditions, breeds, sex, body weights, stages in the feedlot, and diets.

## Introduction

Access to high-quality water is a critical requirement for efficient feedlot production (NRC 2016; Wagner and Engle 2021). The primary demand for water in feedlots is for livestock consumption (78–91% of total use), feed processing (1–6% of total), dust suppression, cleaning and washing cattle and machinery (0–12% of total), and effluent management and other purposes (0–7% of total) (Davis et al. 2008; Duncan 2020). Intake of water is influenced by the weight of cattle and environmental conditions (NRC 2016). A number of measures are used to evaluate environmental effects including mean daily temperatures, maximum, and minimum temperatures, precipitation, humidity, wind speed, atmospheric pressure, solar radiation, and measures that integrate these including heat load index (HLI), accumulated heat load units (AHLU) (Gaughan et al. 2008), temperature humidity index (THI) (Thom 1959), and cattle comfort index (CCI) (Cowan et al. 2024). Water intake in feedlot cattle may also be influenced by the provision of shade (Gaughan et al. 2010; DeBord et al. 2024), dietary salt content (Mader and Davis 2004; Gaughan and Mader 2009), sulfur content of water (Loneragan et al. 2001; Cammack et al. 2010), and other factors including nitrate and metal ions, as well as fecal and algal contamination (NRC 2016; Wagner and Engle 2021). Dry matter intake (Hicks et al. 1988; Arias and Mader 2011; NRC 2016) and possibly the water content of the diet (Bond et al. 1976) may also influence the water intake of feedlot cattle. Machado et al. (2021) provide details on the measures included in a substantial number of studies that produced equations to predict water intake.

The objectives of this study were to undertake a systematic review that evaluated drinking water intake (DWI) of cattle in feedlots or were confined under feedlot conditions only, that is cattle not at pasture and examine the effects of environment and other interventions on DWI. We hypothesized that DWI would reflect the body weight of the cattle and environmental conditions. Meta-analytical models that predict DWI were developed and tested against industry standard predictions (NRC 2016) and against observations from literature. These evaluations were undertaken to assess model efficacy. Further, where appropriate, factors that might influence DWI but having a limited study base in feedlots are reviewed quantitatively and qualitatively. This is the first meta-analysis to evaluate factors that influence DWI of lot fed cattle.

## Materials and methods

As this was a literature-based meta-analysis no animal use permission was required.

We utilized meta-analytical methods described by Lean et al. (2009), Sargeant and O’Connor (2020), and Page et al. (2021) to undertake the review and analyses. The latter are consistent with those of the review by Tempelman (2025).

A comprehensive search of the English language literature was conducted between the 30 June and the 15 July 2025 using the US National Library of Medicine National Institutes of Health database through PubMed (http://www.ncbi.nlm.nih.gov/pubmed), Google Scholar (http://scholar.google.com/), and the ISI Web of Science (http://apps.webofknowledge.com) databases.

The search terms used were as follows, with no limits and were sorted by relevance/best match: (water intake OR water usage OR water consumption OR water requirement) AND (beef feedlot OR finish* cattle OR confine* cattle OR steer OR heifer). The searches were augmented with library searches of relevant journals, examination of cited literature in papers identified and review of citations in review papers.

Citations of the search results were downloaded to Excel (Microsoft Corporation, Microsoft 365, 2025) and duplicates were removed. The search results were pre-screened manually by two reviewers based on citation title and abstract to identify relevant results to proceed to the screening stage. Full texts were also reviewed by two reviewers. As the Google Scholar search yielded 1,420,000 results, the results sorted by relevance were pre-screened by title until there were 20 consecutive results that were off topic. This resulted in pre-screening of 180 titles. Similar screening approaches for Google Scholar have been used by Lean et al. (2021), Malik et al. (2022), and Villoz et al. (2024).

Experiments were included in the analysis if they met the following inclusion criteria: were full manuscripts from peer-reviewed journals published in English language or were peer reviewed theses; experiments were in vivo; the experiments evaluated DWI in feedlot cattle; these had appropriate study designs, both observational and randomized designs; had appropriate analysis of data; contained sufficient data to determine the effect size (ES) for DWI (e.g., the number of cattle or pens in each treatment and control group); and had a measure of variance (SE or SD) for each effect estimate or a *P* value. Experiments that could not be adequately interpreted were excluded. Study designs considered acceptable included observational studies, factorial designs, completely randomized controlled trials, and Latin square trials. We evaluated studies for possible pseudo-replication and included those that were not clearly pseudo-replicated. Blinding was not appropriate to assess as many studies were observational or clearly evident to those involved. Appropriate statistical evaluation was assessed to ensure that the analysis methods used were consistent with the study design, noting that some older studies used analytical methods that may be superseded. As these data were a mixture of observational data and intervention studies the most appropriate means of evaluation was to use a mixed models meta-regression approach (St-Pierre 2001).

**Figure 1:**
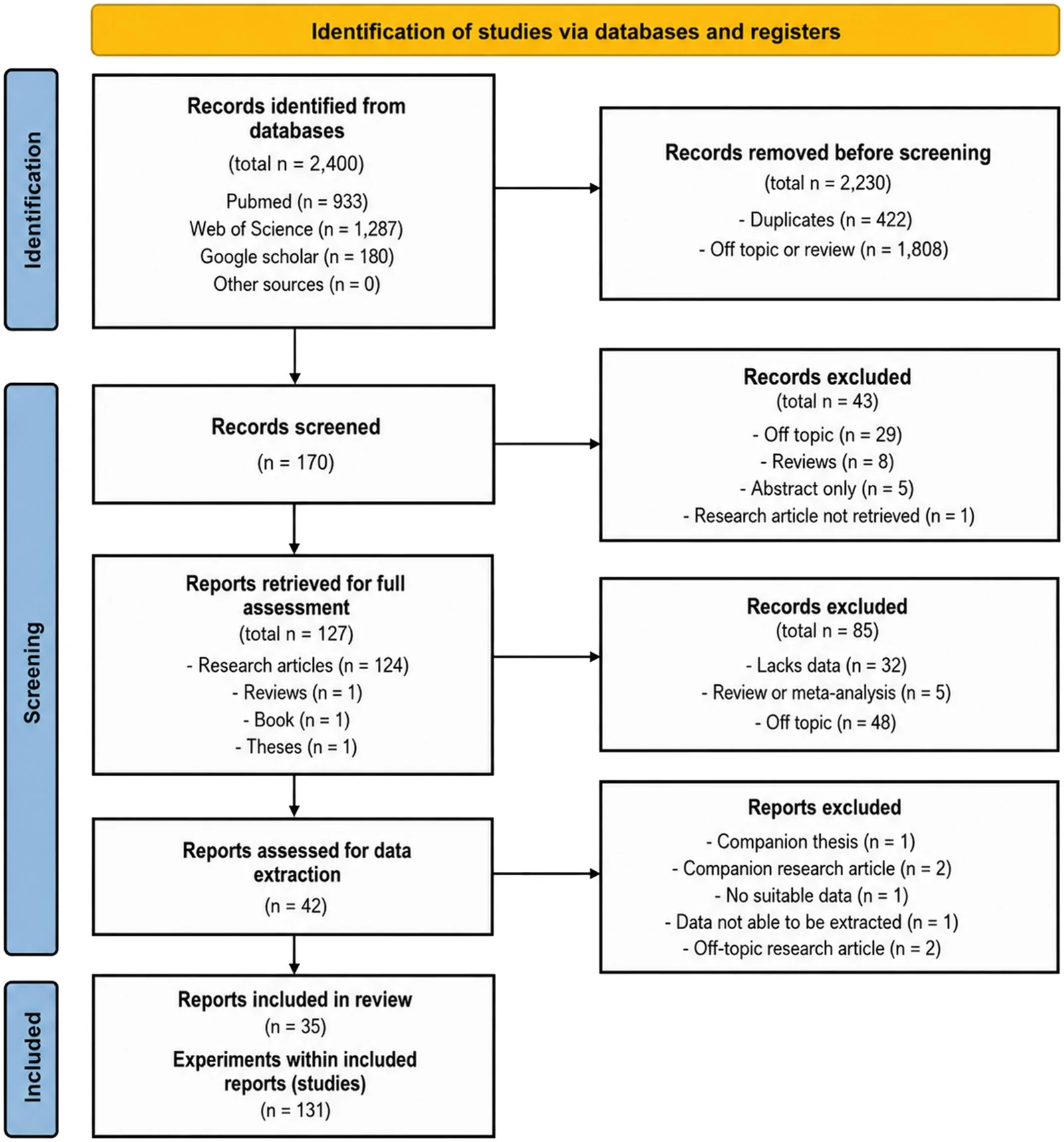
PRISMA flow diagram (adapted from Page et al. [2021]) of the systematic review from initial search and screening to final selection of publications to be included in the meta-analysis on water intake in feedlot cattle. The *n* refers to the number of records for identification and screening that includes experimental articles, abstracts, books, review papers, theses, patents, and other records.

Figure 1 depicts a PRISMA diagram (Page et al. 2021) of the flow of data collection for the meta-analysis. The search identified 2,400 records, of which 2,230 were excluded before screening as they were reviews or off topic (*n* = 1,808) or duplicates (*n* = 422). Of the 170 records that reached screening, only 42 reports reached data extraction.

One study (Moehlenpah et al. 2021) met all the inclusion criteria but appeared to be conducted with lactating cows and was excluded. A total of 35 reports or studies with up to 131 experimental comparisons were included in the meta-analysis. For one study, data were sourced from the two companion papers, Ahlberg et al. (2018) and Ahlberg et al. (2019). A list of articles excluded at the final data extraction step with the reason is provided in Table S1. Decision rules were used to account for the variability in reporting from the studies as follows. Bodyweight, temperature, humidity, and other environmental effects were assessed whenever possible for the period that best approximated the interval over which DWI was recorded. Where an initial and finishing body weight was provided body weight was estimated as the mean of these to match the interval when temperature measures were made. Numbers used for control or treatment in an experiment were based on the number of pens or cattle depending on the unit of interest. Four estimates of variance were derived from *P* values based on the critical value of the *t*-distribution.

Data were extracted from each of the experiments that met the inclusion criteria and were audited by three reviewers. The descriptive data extracted included aspects of experimental design and details about the cattle used. Design details included the country in which the study was conducted, the system used to measure DWI, whether randomization was stated, possible pseudo-replication where insufficient detail was provided to determine occurrence of pseudo-replication, the study design, breed, sex, stage of feeding (background, lot, or mixture) the number of experimental units per control and treatment, the experimental unit (individual animal or pen), age of cattle and days over which treatment effects were measured, type of control and intervention (if used), whether cattle were vaccinated, dewormed or hormonal growth promotants were used, the number of days on trial, temperature as reported in the paper or averaged over the days on trial, humidity, THI, wind speed, shade, season, salt concentration of feed and drinking water, sulfur concentration of feed and drinking water, body weight of cattle, average daily gain, dry matter intake (DMI) and dry matter content of the feed as indicated in the article or estimated from the ingredients in the diet (Table 1). The temperature measure used in this study is best considered to be average daily temperature. Data were only analyzed for outcomes that had *n* ≥ 3 studies.

**Table 1:** Descriptives of studies and experiments included.

Author	Country	Design	Breed	Sex	Stage	Pen or I	Head (*n*)	Analysis (*n*) cattle or pen	DWI (L/d)	Temp (°C)	Shade	Experiment description
**Ahlberg et al. (2018 and 2019)**	USA	Obs	Mixed	Steer	FL	Pen	117	4	40.5	25.0	Yes	Summer 2014
**Ahlberg et al. (2018 and 2019)**	USA	Obs	Mixed	Steer	FL	Pen	116	4	28.2	4.0	Yes	Winter 2014
**Ahlberg et al. (2018 and 2019)**	USA	Obs	Mixed	Steer	FL	Pen	118	4	36.4	23.4	Yes	Summer 2015
**Ahlberg et al. (2018 and 2019)**	USA	Obs	Mixed	Steer	FL	Pen	105	4	49.5	28.1	Yes	Summer 2016
**Ahlberg et al. (2018 and 2019)**	USA	Obs	Mixed	Steer	FL	Pen	123	4	34.9	9.7	Yes	Winter 2017
**Allwardt et al. (2017)**	USA	Obs	Crossbred	Steer	FL	I	117	117	25.2		Yes	*Ad libitum* water intake
**Arias and Mader (2011)**	USA	Obs	Mixed	Mixed	FL	Pen	144	7	24.6	9.3	No	Overall (combined seasons)
**Arias and Mader (2023)**	USA	Obs	Angus cross	Steer	FL	Pen	16	16	23.6		No	Overall model: May–October
**Arias and Mader (2023)**	USA	Obs	Angus cross	Steer	FL	Pen	16	16	34.2	24.2	No	Summer model: July–August
**Beaver et al. (1989)**	USA	Fac^1^	Angus	Steer	BG	I	4	1	8.80	18.0	Yes	Exp 1: Angus, 18 °C, growing
**Beaver et al. (1989)**	USA	Fac^1^	Brangus	Steer	BG	I	4	1	4.80	18.0	Yes	Exp 1: Brangus, 18 °C, growing
**Beaver et al. (1989)**	USA	Fac^1^	Angus	Steer	BG	I	4	1	10.0	30.0	Yes	Exp 1: Angus, 30 °C, growing
**Beaver et al. (1989)**	USA	Fac^1^	Brangus	Steer	BG	I	4	1	6.40	30.0	Yes	Exp 1: Brangus, 30 °C, growing
**Beaver et al. (1989)**	USA	Fac^1^	Angus	Steer	BG	I	2	1	12.6	18.0	Yes	Exp 2: Angus, 18 °C, growing
**Beaver et al. (1989)**	USA	Fac^1^	Brangus	Steer	BG	I	2	1	7.60	18.0	Yes	Exp 2: Brangus, 18 °C, growing
**Beaver et al. (1989)**	USA	Fac^1^	Angus	Steer	BG	I	2	1	44.0	30.0	Yes	Exp 2: Angus, 30 °C, growing
**Beaver et al. (1989)**	USA	Fac^1^	Brangus	Steer	BG	I	2	1	28.2	30.0	Yes	Exp 2: Brangus, 30 °C, growing
**Beaver et al. (1989)**	USA	Fac^1^	Angus	Steer	FL	I	2	1	17.2	18.0	Yes	Exp 3: Angus, 18 °C, finishing
**Beaver et al. (1989)**	USA	Fac^1^	Brangus	Steer	FL	I	2	1	7.50	18.0	Yes	Exp 3: Brangus, 18 °C, finishing
**Beaver et al. (1989)**	USA	Fac^1^	Angus	Steer	FL	I	2	1	51.2	33.0	Yes	Exp 3: Angus, 33 °C, finishing
**Beaver et al (1989)**	USA	Fac^1^	Brangus	Steer	FL	I	2	1	19.8	33.0	Yes	Exp 3: Brangus, 33 °C, finishing
**Bond et al. (1976)**	USA	LS	European	Steer	FL	I	6	6	44.5		Yes	0% forage diet
**Bond et al. (1976)**	USA	LS	European	Steer	FL	I	6	6	41.4		Yes	33% forage diet
**Bond et al. (1976)**	USA	LS	European	Steer	FL	I	6	6	67.0		Yes	88% forage diet
**Cammack et al. (2010)**	USA	RCT	NS	Steer	FL	Pen	24	3	49.4		NS	Water sulfate: low
**Cammack et al. (2010)**	USA	RCT	NS	Steer	FL	Pen	24	3	42.1		NS	Water sulfate: high
**Cammack et al. (2010)**	USA	RCT	NS	Steer	FL	Pen	24	3	42.4		NS	Water sulfate: high + 2.5% clinoptilolite
**Cammack et al. (2010)**	USA	RCT	NS	Steer	FL	Pen	24	3	44.6		NS	Water sulfate: high + 5% clinoptilolite
**da Silva et al. (2025)**	USA	RCT	Angus cross	Steer	BG	I	12	12	36.5		Yes	Moderate BGer diet
**da Silva et al. (2025)**	USA	RCT	Angus cross	Steer	BG	I	12	12	38.6		Yes	High BGer diet
**da Silva et al. (2025)**	USA	RCT	Angus cross	Steer	FL	I	12	12	40.8		Yes	Grain finisher diet
**da Silva et al. (2025)**	USA	RCT	Angus cross	Steer	FL	I	12	12	70.0		Yes	Forage finisher diet
**DeBord et al. (2024)**	USA	RCT	Angus cross	Heifer	FL	Pen	213	20	44.9	26.6	No	No shade: high roughage *ad lib*
**DeBord et al. (2024)**	USA	RCT	Angus cross	Heifer	FL	Pen	213	20	39.8	26.6	No	No shade: high energy limit fed
**DeBord et al. (2024)**	USA	RCT	Angus cross	Heifer	FL	Pen	213	20	40.0	26.6	Yes	Shaded: high roughage *ad lib* diet
**DeBord et al. (2024)**	USA	RCT	Angus cross	Heifer	FL	Pen	213	20	34.7	26.6	Yes	Shaded: high energy limit fed
**Digesti and Weeth (1976)**	USA	RCT	Hereford × Angus	Heifer	BG	I	4	4	38.5		Yes	Water sulfate: control (Tap): 110 mg/L
**Digesti and Weeth (1976)**	USA	RCT	Hereford × Angus	Heifer	BG	I	4	4	31.5		Yes	Water sulfate: 1250 mg/L
**Digesti and Weeth (1976)**	USA	RCT	Hereford × Angus	Heifer	BG	I	4	4	33.1		Yes	Water sulfate: 2,500 mg/L
**Gaughan and Mader (2009)**	USA	LS	Crossbred	Mixed	FL	Pen	48	18	31.8	26.1	NS	Exp 1: summer control: no dietary salt or soy
**Gaughan and Mader (2009)**	USA	LS	Crossbred	Mixed	FL	Pen	48	18	32.9	26.1	NS	Exp 1: summer: dietary salt
**Gaughan and Mader (2009)**	USA	LS	Crossbred	Mixed	FL	Pen	48	18	31.8	26.1	NS	Exp 1: summer: dietary salt + soy
**Gaughan and Mader (2009)**	USA	LS	Crossbred	Mixed	FL	Pen	42	24	16.8	−2.9	NS	Exp 2: winter control: no dietary salt or soy
**Gaughan and Mader (2009)**	USA	LS	Crossbred	Mixed	FL	Pen	42	24	19.1	−2.9	NS	Exp 2: winter: dietary salt
**Gaughan and Mader (2009)**	USA	LS	Crossbred	Mixed	FL	Pen	42	24	17.2	−2.9	NS	Exp 2: winter: dietary soy
**Gaughan and Mader (2009)**	AU	LS	Crossbred	Mixed	FL	Pen	42	24	18.8	−2.9	NS	Exp 2: winter: dietary salt + soy
**Gaughan and Mader (2009)**	AU	Fac	Angus	Steer	FL	I	12	4	18.2	21.6	Yes	Exp 3: thermoneutral control: no dietary salt or fat
**Gaughan and Mader (2009)**	AU	Fac	Angus	Steer	FL	I	12	4	20.1	21.6	Yes	Exp 3: thermoneutral: dietary salt
**Gaughan and Mader (2009)**	AU	Fac	Angus	Steer	FL	I	12	4	22.8	21.6	Yes	Exp 3: thermoneutral: dietary salt + fat
**Gaughan and Mader (2009)**	AU	Fac	Angus	Steer	FL	I	12	4	39.0	29.9	Yes	Exp 3: hot control: no dietary salt or fat
**Gaughan and Mader (2009)**	AU	Fac	Angus	Steer	FL	I	12	4	40.6	29.9	Yes	Exp 3: hot: dietary salt
**Gaughan and Mader (2009)**	AU	Fac	Angus	Steer	FL	I	12	4	46.1	29.9	Yes	Exp 3: hot: dietary salt + fat
**Gaughan et al. (2010)**	AU	RCT	Angus	Steer	FL	Pen	16	10	53.1	25.6	No	No shade
**Gaughan et al. (2010)**	IT	RCT	Angus	Steer	FL	Pen	16	10	49.3	25.6	Yes	Shade
**Grossi et al. (2021)**	IT	RCT	Charolais	Bull	BG	Pen	112	16	30.1		Yes	Warm water
**Grossi et al. (2021)**	USA	RCT	Charolais	Bull	BG	Pen	112	16	25.9		Yes	Room temperature water
**Grout et al. (2006)**	USA	LS	Hereford, Hereford × Angus	Mixed	BG	I	16	4	42.7	28.7	Yes	Control
**Grout et al. (2006)**	USA	LS	Hereford, Hereford × Angus	Mixed	BG	I	16	4	38.9	28.7	Yes	Water sulfate: low (Na_2_SO_4_)
**Grout et al. (2006)**	USA	LS	Hereford, Hereford × Angus	Mixed	BG	I	16	4	39.7	28.7	Yes	Water sulfate: high (Na_2_SO_4_)
**Grout et al. (2006)**	KR	LS	Hereford, Hereford × Angus	Mixed	BG	I	16	4	29.7	28.7	Yes	Water sulfate: higher (Na_2_SO_4_)
**Kim et al. (2023)**	KR	RCT	KRn native	Steer	BG	I	12	4	45.9		Yes	Low THI
**Kim et al. (2023)**	KR	RCT	KRn native	Steer	BG	I	12	4	45.8		Yes	Medium THI
**Kim et al. (2023)**	SP	RCT	KRn native	Steer	BG	I	12	4	63.2		Yes	High THI
**Llonch et al. (2020)**	SP	LS	Simmental	Heifer	FL	I	8	2	26.2		Yes	6.4% peNDF TMR
**Llonch et al. (2020)**	SP	LS	Simmental	Heifer	FL	I	8	2	24.6		Yes	10.4% peNDF TMR
**Llonch et al. (2020)**	SP	LS	Simmental	Heifer	FL	I	8	2	25.5		Yes	13.6% peND TMR
**Llonch et al. (2020)**	SP	LS	Simmental	Heifer	FL	I	8	2	24.4		Yes	15.4% peNDF TMR
**Llonch et al. (2023)**	SP	RCT	Holstein	Bull	FL	I	6	6	37.0		Yes	Control: in the 210 d of 1st study
**Llonch et al. (2023)**	SP	RCT	Holstein	Bull	FL	I	6	6	36.0		Yes	Acid water: in the 210 d of 1st study
**Llonch et al. (2023)**	SP	RCT	Holstein	Bull	FL	I	6	6	33.4		Yes	Cl water: in the 210 d of 1st study
**Llonch et al. (2023)**	SP	RCT	Holstein	Bull	FL	I	6	6	35.9		Yes	Acid + Cl: in the 210 d of 1st study
**Llonch et al. (2023)**	SP	RCT	Holstein	Bull	FL	Pen	49	3	39.4	22.1	Yes	Control: in the 112 d of 2nd study
**Llonch et al. (2023)**	USA	RCT	Holstein	Bull	FL	Pen	47	3	43.5	22.1	Yes	Acid + Cl: in the 112 d of 2nd study
**Lofgreen et al. (1975)**	USA	RCT^1^	British, Brahman × British	Mixed	FL	Pen	60	3	37.1	33.0	Yes	Water 32.2 ^o^C
**Lofgreen et al. (1975)**	USA	RCT^1^	British, Brahman × British	Mixed	FL	Pen	60	3	35.2	33.0	Yes	Water 18.3 ^o^C
**Loneragan et al. (2001)**	USA	RCT	Crossbred	Steer	FL	Pen	24	3	34.4	20.1	NS	Water sulfate: 136 mg/L
**Loneragan et al. (2001)**	USA	RCT	Crossbred	Steer	FL	Pen	24	3	35.0	20.1	NS	Water sulfate: 291 mg/L
**Loneragan et al. (2001)**	USA	RCT	Crossbred	Steer	FL	Pen	24	3	32.0	20.1	NS	Water sulfate: 583 mg/L
**Loneragan et al. (2001)**	USA	RCT	Crossbred	Steer	FL	Pen	24	3	32.4	20.1	NS	Water sulfate: 1,219 mg/L
**Loneragan et al. (2001)**	BR	RCT	Crossbred	Steer	FL	Pen	24	3	30.1	20.1	NS	Water sulfate: 2,360 mg/L
**Machado et al. (2021)**	BR	Obs	Brangus	Heifer	FL	Pen	20	3	12.7	19.5	NS	Year 1
**Machado et al. (2021)**	USA	Obs	Brangus	Heifer	FL	Pen	10	3	11.5	14.7	NS	Year 2
**Mader and Davis (2004)**	USA	RCT	Angus × Charolais	Steer	FL	Pen	48	3	41.1	22.1	NS	Exp 1: Ad lib fed
**Mader and Davis (2004)**	USA	RCT	Angus × Charolais	Steer	FL	Pen	48	3	43.5	22.1	NS	Exp 1: Slick bunk
**Mader and Davis (2004)**	USA	RCT	Angus × Charolais	Steer	FL	Pen	48	3	35.0	22.1	NS	Exp 1: Limit fed
**Mader and Davis (2004)**	USA	RCT	Angus × Charolais	Steer	FL	Pen	24	2	38.0	22.1	NS	Exp 2: Control: no sprinkle
**Mader and Davis (2004)**	USA	RCT	Angus × Charolais	Steer	FL	Pen	24	2	37.7	22.1	NS	Exp 2: Sprinkle am
**Mader and Davis (2004)**	USA	RCT	Angus × Charolais	Steer	FL	Pen	24	2	40.1	22.1	NS	Exp 2: Sprinkle pm
**Mader et al. (2010)**	USA	RCT	Angus cross	Steer	FL	Pen	42	6	30.4	23.1	NS	Control
**Mader et al. (2010)**	USA	RCT	Angus cross	Steer	FL	Pen	42	6	37.1	23.1	NS	Dietary KCO_3_
**Mader et al. (2010)**	USA	RCT	Angus cross	Steer	FL	Pen	42	6	26.8	23.1	NS	Dietary NaCl
**Mader et al. (2010)**	BR	RCT	Angus cross	Steer	FL	Pen	42	6	38.0	23.1	NS	Dietary KCO_3_ + NaCl
**Maia et al. (2023)**	BR	RCT	Nellore and cross	Bull	FL	Pen	400	8	36.1	25.9	No	No shade
**Maia et al. (2023)**	BR	RCT	Nellore and cross	Bull	FL	Pen	400	8	34.9	25.9	Yes	Shade
Novelli et al. (2022)	BR	RCT	Nellore	Bull	FL	I	23	23	40.1	23.0	No	No shade
Novelli et al. (2022)	CA	RCT	Nellore	Bull	FL	I	23	23	36.8	23.0	Yes	Shade
Penner et al. (2020)	CA	RCT	Hereford cross	Heifer	BG	I	16	8	26.0		Yes	Water sulfate: 292 mg/L (control)
Penner et al. (2020)	CA	RCT	Hereford cross	Heifer	BG	I	16	8	24.7		Yes	Water sulfate: 1245 mg/L
Penner et al. (2020)	CA	RCT	Hereford cross	Heifer	BG	I	16	8	26.4		Yes	Water sulfate: 2,305 mg/L
Penner et al. (2020)	BR	RCT	Hereford cross	Heifer	BG	I	16	8	24.6		Yes	Water sulfate: 3,376 mg/L
Pires et al. (2022)	BR	Obs	Caracu	Bull	FL	I	61	61	21.2		NS	Male
Pires et al. (2022)	USA	Obs	Caracu	Heifer	FL	I	43	43	19.4		NS	Female
Ray (1989)	USA	Obs	Crossbred	Steer	Mix	Pen	32	32	32.1	35.5	Yes	Summer
Ray (1989)	USA	Obs	Crossbred	Steer	Mix	Pen	32	32	27.9	25.1	Yes	Winter
Ray (1989)	USA	RCT	Crossbred	Steer	Mix	Pen	32	32	33.2	30.3	Yes	BG
Ray (1989)	USA	RCT	Crossbred	Steer	Mix	Pen	32	32	26.7	30.3	Yes	Lot
Ray (1989)	USA	RCT	Crossbred	Steer	Mix	Pen	16	16	29.3	30.3	Yes	Control: (normal water in both periods)
Ray (1989)	USA	RCT	Crossbred	Steer	Mix	Pen	16	16	29.6	30.3	Yes	Control then saline water
Ray (1989)	USA	RCT	Crossbred	Steer	Mix	Pen	16	16	29.8	30.3	Yes	Saline water then control
Ray (1989)	USA	RCT	Crossbred	Steer	Mix	Pen	16	16	31.3	30.3	Yes	Saline water then saline water again
Ray (1989)	USA	Fac	Crossbred	Steer	Mix	Pen	20	8	29.3		Yes	Normal water 1,300 ppm TDS
Ray (1989)	USA	Fac	Crossbred	Steer	Mix	Pen	20	8	29.6		Yes	Normal then saline water 6,000 ppm TDS
Ray (1989)	USA	Fac	Crossbred	Steer	Mix	Pen	20	8	29.8		Yes	Saline water 6,000 ppm TDS then Normal
Ray (1989)	USA	Fac	Crossbred	Steer	Mix	Pen	20	8	31.3		Yes	Saline water 6,000 ppm TDS
Ray (1989)	USA	Fac	Crossbred	Steer	Mix	Pen	10	16	32.1	26.3	Yes	Summer year 2
Ray (1989)	USA	Fac	Crossbred	Steer	Mix	Pen	10	16	27.9	16.5	Yes	Winter year 2
Sexson et al. (2010)	USA	RCT	Cross bred	Steer	FL	Pen	48	4	37.5		No	Control: reverse osmosis + well water
Sexson et al. (2010)	USA	RCT	Crossbred	Steer	FL	Pen	48	4	34.7		No	Well water
Sexson et al. (2010)	USA	RCT	Crossbred	Steer	FL	Pen	48	4	37.2		No	Dietary 0.75% potassium chloride
Sexson et al. (2010)	USA	RCT	Crossbred	Steer	FL	Pen	48	4	34.8		No	Dietary 0.75% potassium carbonate
Sexson et al. (2010)	USA	RCT	Crossbred	Steer	FL	Pen	48	4	36.4		No	Dietary 1.0% potassium carbonate
Sexson et al. (2012)	AU	Obs	NS	Steer	FL	Pen	75	75	37.1	22.0	No	Observational
Sullivan et al. (2011)	AU	RCT	Angus	Heifer	FL	Pen	18	2	43.0	24.1	No	No shade
Sullivan et al. (2011)	AU	RCT	Angus	Heifer	FL	Pen	36	4	38.0	24.1	Yes	2 m shade
Sullivan et al. (2011)	AU	RCT	Angus	Heifer	FL	Pen	38	4	38.0	24.1	Yes	3.3 m shade
Sullivan et al. (2011)	AU	RCT	Angus	Heifer	FL	Pen	36	4	38.0	24.1	Yes	4.7 m shade
Sullivan et al. (2022)	USA	Obs	Angus	Steer	FL	I	18	18	56.6	29.9	Yes	Climate room: thermoneutral
Treon et al. (2024)	USA	RCT	Angus cross	Steer	FL	Pen	20	2	16.3		Yes	Control: no dietary probiotics
Treon et al. (2024)	USA	RCT	Angus cross	Steer	FL	Pen	20	2	15.1		Yes	Dietary probiotics
Volpi-Lagreca and Duckett (2016)	USA	Obs	NS	Steer	FL	Pen	12	3	36.6		NS	Control: no additives
Wijffels et al. (2024)	AU	Obs	Angus	Steer	FL	I	12	12	67.1	29.9	Yes	Pre-challenge thermoneutral

Obs = observational study; Fac = factorial; RCT = randomized controlled trial; LS = Latin square; I = individual; NS = not stated; DWI, drinking water intake; Temp = temperature; THI = temperature humidity index; FL = feedlot and BG = Backgrounding and all the abbreviations for country that were added: USA = United States of America, AU = Australia, IT = Italy, BR = Brazil, CA = Canada, KOR = Korea, SP = Spain.

1 Insufficient detail was provided to determine whether pseudoreplication had occurred.

For THI, a calculation was made for studies (29 experiments) that reported temperature, humidity, and wind speed but did not report THI based on Thom (1959) where THI = (0.8 Average temperature) + [(Relative humidity/100)*(average temperature − 14.4)] + 46.4. The HLI was calculated for 32 experiments according to Gaughan et al. (2008) as HLI = 8.62 + 0.38*relative humidity + 1.55*black globe temperature + e^(−windspeed*2.4)^ −0.5*windspeed with the assumption being to use the model for temperatures >25 °C. Use of average daily temperature will underestimate the HLI; however, very few studies reported black globe values. Data were not available to calculate either AHLU or CCI.

### Statistical analysis

All data analysis was performed in Stata (Version 19, StataCorp. LP, College Station, TX). Initial data exploration included production of basic statistics to examine the data for possible errors and to estimate the means and measures of dispersion. Normality of the data was examined by visual and statistical appraisal for continuous variables.

The following production outcomes were suitable for analysis: DWI (L/d), average daily gain (ADG) (kg/d), and DMI (kg/d). Factors that were evaluated as potentially influencing DWI included breed of cattle, sex, body weight of cattle, temperature, humidity, THI, HLI, whether cattle were shaded, sulfur content of water, ADG, DMI, and diet dry matter (DM) content.

Studies obtained through the literature search included both observational studies, experimental multiarm studies, factorial designs, and Latin square designs. Consequently, the optimal statistical approach was to use a mixed model meta-regression (Stata *meta meregress*) that allowed for the random effects of experiment within study. The covariance structure used was independent reflecting the data structure and potential to test for random slopes. Only random effects models were used, as previous work concluded that when there was uncertainty in the evaluative units caused by clustering of observations, the random effects model was appropriate (White and Thomas 2005). The number of experiments that examined different factors that might influence DWI varied and given the nesting of experiments within studies (multiarm studies), there were very few independent observations associated with some outcomes (Tables 1 and 2). Consequently, the model development process was focused on developing a more generalizable model. Less frequently studied outcomes were evaluated in univariable multilevel models but not multivariable multilevel models. Collinearity and correlations among body weight, ADG, DMI, diet DM, and temperature and THI were assessed using Stata *collin* and *spearman*, respectively. The multivariable, multilevel models were constructed by backward stepping separately for temperature and THI. Interactions among variables were tested and quadratic effects of temperature and body weight were tested. Neither interactions nor quadratic terms entered (*P* > 0.1) final models. Evaluation of residuals from models (Stata *predict xb1, standard*) lead to an evaluation of a reduced dataset with experimental temperatures of <12 °C (*n* = 7) excluded from the final model as outlying residuals were clustered for temperatures of <12 °C. Residuals from the two random effects were evaluated (Stata *predict xb2 xb3*) and were acceptably distributed. A sensitivity analysis was conducted to evaluate whether large or influential studies or study design influenced results. Statistical confounding was assessed as present if the co-efficient of the predictor variable changed by more than 25% (Rothman 1986). A pseudo-*R*^2^ was calculated at the overall level and for study and experiment level models by calculating the true variance, tau-squared (τ^2^) at overall and at each level; the pseudo-*R*^2^ overall and at each level = 1 – (τ^2^ test model or level/τ^2^ for the null model or level). A similar method of analysis was used to evaluate a model that contained THI rather than temperature.

**Table 2:** Descriptive statistics for reported and calculated variables at the experiment level.

Measures	*N* ^1^	Mean	SD	Median	Minimum	Maximum
**Total number of cattle used**	131	219	735.5	96	4	8,209
**Number of head per group**	131	40.5	62.17	4	2	400
**Number used in analysis**	131	10.1	14.43	20	1	117
**Water, L/d**	131	33.2	11.90	34.7	4.80	70.0
**Water SD, L/d**	129	4.3	8.95	2.95	0.12	100
**Days in trial**	131	94.3	130.68	85.0	5.00	1460
**Days of recording**	131	79.2	132.76	70.0	5.00	1460
**Temperature, ^o^C**	84	23.1	8.02	24.1	−2.90	35.5
**Relative humidity, %**	58	62.6	12.86	68.0	32.7	81.5
**Temperature humidity index**	68	69.1	12.11	72.2	31.3	83.5
**Wind speed, m/s**	37	4.74	3.79	2.67	1.78	12.7
**Heat load index**	32	70.2	9.62	72.4	40.6	84.2
**Body weight, kg**	125	382.7	121.10	358.0	195.0	712.9
**Body weight SD, kg**	82	16.9	12.75	16.4	2.10	70.2
**Average daily gain, kg/d**	76	1.32	0.42	1.34	0.33	2.16
**Average daily gain SD, kg/d**	74	0.30	0.25	0.25	0.05	1.41
**Dry matter intake, kg**	114	8.98	2.97	9.16	3.50	21.3
**Dry matter intake SD, kg**	108	0.70	0.50	0.54	0.05	1.78
**Diet dry matter, %**	111	79.8	13.63	88.0	38.8	93.9

1 Number of experimental comparisons.

A key focus of meta-analysis is to identify and understand the sources of heterogeneity or variation of response among comparisons. Heterogeneity of results among the study and also experimental comparisons were quantified using the *I*^2^ statistic (Higgins and Thompson 2002) and were reported as the total estimates from the study and experiment levels using the total study *I*^2^ Higgins–Thompson estimation using Stata *estat heterogeneity* based on methods developed by Nakagawa and Santos (2012). The *estat heterogeneity* command provides estimates of heterogeneity at the study and experiment level.

The *I*^2^ provides an estimate of the proportion of the true variance of effects of the treatment, that is the true variance, τ^2^ divided by the total variance observed in the comparison (Borenstein et al. 2017) that reflect measurement error. Negative values of *I* are assigned a value of 0, consequently the value *I*^2^ lies between 0% and 100%. An *I*^2^ value between 0% and 40% might not be important, 30–60% may represent moderate heterogeneity, 50–90% might represent substantial heterogeneity, and 75–100% might represent considerable heterogeneity (Higgins and Green 2011).

For evaluation of the models, data were extracted from NRC (2016) for finishing cattle that were based on Winchester and Morris (1956). These were tested against estimations of DWI for temperatures over 12°C in the model developed from the data in this study. Lin’s concordance correlation coefficient *ρ*_c_ (Lin 1989), Pearson’s correlation, and Bland and Altman’s (1986) limits of agreement were calculated and graphs were produced (Stata *concord*).

Differences in DWI responses to sulfur content of the water were evaluated by standardized mean difference (SMD) [Stata *esize(mdiff*)] as these were suitable to evaluate using classical meta-analytical methods. The SMD estimates were pooled using the DerSimonian and Laird (1986) random effects models (D&L). As experiments were nested within studies, Stata *meta multilevel* (Goldstein et al. 2000; Thompson et al. 2001) was used to provide a more correct measure of the SMD using the units appropriate to the single level analysis with both the single level and multilevel effects being presented using Stata *meta forestplot*.

A key focus of meta-analysis is to identify and understand the sources of heterogeneity or variation of response among comparisons. This was investigated by evaluating the influence of difference in water sulfur content between treatment and controls. The effects of one experiment with a very large difference in water sulfur, Llonch et al. (2023) was investigated using the Stata *meta summarize leaveoneout* option.

Data were evaluated for potential publication bias using *meta funnelplot* with the contours of significance identified. Funnel plots are a simple scatter plot of the intervention effect estimates from individual comparisons plotted against comparison precision. The name “*funnel plot*” arises because precision of the intervention effect increases as the size and precision of a comparison increases. Effect estimates from comparisons with a small number of animal units will scatter more widely at the bottom of the graph and the spread narrows for those with higher numbers of units. In the absence of bias, the plot should approximately resemble a symmetrical (inverted) funnel.

## Results and discussion

A total of 131 experiments nested within 35 different studies (for one study data was sourced from two companion papers) were included in the dataset but some experiments (*n* = 19) were observational, and many evaluated different factors influencing DWI thereby influencing the selection of variables in a mixed model meta-analytical approach (Table 1).

Twenty-three studies and 90 experiments were in the United States, 5 studies and 14 experiments were in Australia, 4 studies and 8 experiments were in Brazil, 1 study with 4 experiments was in Canada, 1 study with 2 experiments was in Italy, 1 study and 3 experiments was in Korea, and 2 studies and 10 experiments were in Spain (Table 1). As some studies contained mixed sexes, there were 13 experiments with bulls, 22 with heifers, 14 with mixed sexes, and 82 with steers (Table 1).

Breed descriptions were not clearly defined in many studies and experiments (Table 1). Angus or Angus cross listed first in the cross, were primarily used (*n* = 42 experiments), followed by a mix of breeds or crossbreds where the breeds crossed were not reported (*n* = 38 experiments). Hereford or Hereford cross, with Hereford listed first in the cross made up the third largest portion of the experiments (*n* = 11 experiments). Only six experiments did not state the breed used, while the remainder were less frequently reported breeds.

There were 3 studies and 24 experiments that were factorial, 4 studies and 18 experiments that were Latin Squares, 11 studies and 19 experiments that were observational, and 18 studies and 70 experiments that were randomized controlled trials (Table 1). Most studies (*n* = 28) and experiments (*n* = 91) were during the feedlot stage of feeding, while both backgrounding and feedlot stages were evaluated in 2 studies and 14 experiments, and backgrounding only in 7 studies and 26 experiments (Table 1). Shade was used in 24 studies and 85 experiments. A total of 15 studies and 54 experiments were on individual animal intakes of water and 22 studies, and 77 experiments evaluated water intake at the pen level (Table 1). The mean number of head in studies was 219 but ranged from 4 to 8,209 (Table 2).

The mean DWI was 31.2 L/d (SD = 4.31) and mean temperature was 23.1°C (SD = 24.10), and THI was 69.1 (SD = 9.62) at the experiment level (Table 2). The temperature was determined based on the mean average daily temperature reported for short experimental periods; however, the mean average daily temperature was used for longer experiments during which there was often a marked difference in temperature that varied over seasons and day. The average maximum daily temperature was 35.5°C (Table 2); however, greater temperatures were reported in the experiments over shorter times. The mean body weight of 382.7 kg (SD = 16.94) suggests lighter body weight feedlot cattle and the mean DMI of 8.98 kg/d (SD = 0.70) is consistent with those anticipated for lighter feedlot cattle (Table 2). The mean ADG of 1.32 kg/d (SD = 0.30) is consistent with feedlot performance. The wide range of study approaches, countries in which studies were conducted, breeds, sex, and body weight of cattle, variation in interventions used, variation in environmental conditions, and housing and diets (Tables 1 and 2) provide a study base that is likely to be generalizable to the feedlot production environment.

**Figure 2:**
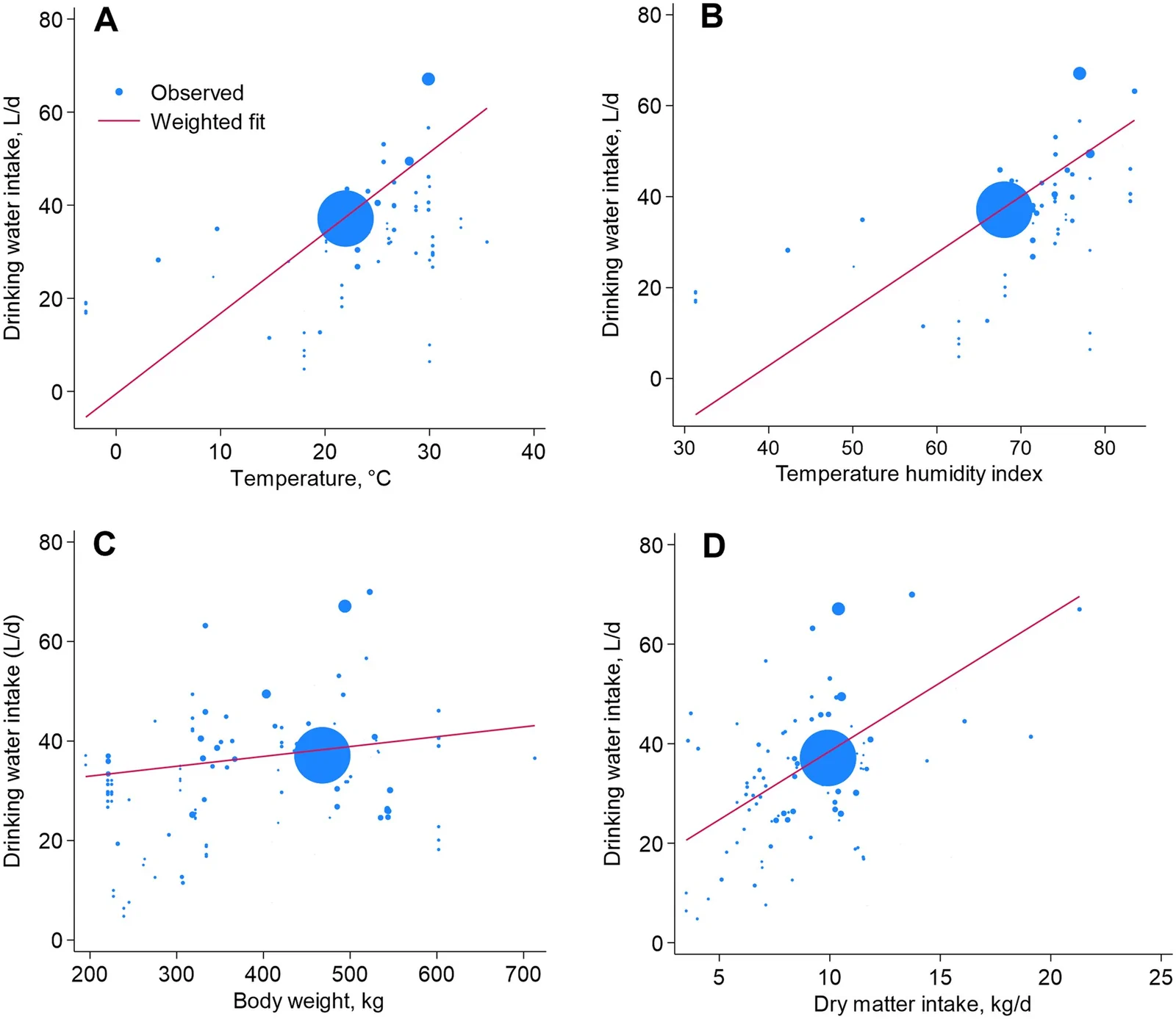
The relationship between drinking water intake (L/d) and A) temperature (°C), B) temperature humidity index, C) body weight (kg), and D) dry matter intake for the experiments. The circular markers indicate the experiment and experiment weight, the larger the marker the greater the weight. The regression line is linear and weighted.

Figure 2A shows the relationship between DWI (L/d) and temperature for the experiments with a regression weighted by effect size which aligns with the findings of Arias and Mader (2011). The information in our data set is sparse for temperatures <15 °C; a finding consistent with 50% of studies evaluating DWI at temperatures consistent with thermal stress (>24 °C). The relationship between DWI and temperature is well identified and forms the basis for care, management, and planning for feedlot cattle (Winchester and Morris 1956; Meat and Livestock Australia 2012; Animal Health Australia 2014; NRC 2016; Wagner and Engle 2021).

The relationship between DWI (L/d) and THI for the experiments with a regression weighted by effect size in Figure 2B is well established (Arias and Mader 2011). The THI is strongly related to core body temperature, rumen temperature, and DMI once a threshold temperature has been reached (Wijffels et al. 2024). Wijffels et al. (2024) citing the work of others (Scharf et al. 2011; Curtis et al. 2017) suggested a threshold THI value of 76 and 25°C average daily temperature beyond which there is an elevation of core temperature for *Bos taurus* feedlot cattle.

Figure 2C shows the relationship between DWI (L/d) and body weight (kg) for the experiments and demonstrates a good range of body weights (195 minimum to 713 kg maximum) in the different experiments (Table 2). As with THI and temperature, the relationship between body weight and DWI (Figure 4) is a well described factor influencing DWI (Winchester and Morris 1956; NRC 2016).

A strong relationship was identified between DWI (L/d) and DMI (kg) for the experiments (Figure 2D). While univariably significant within the multilevel model (Table 3 and Figure 2D), DMI was not significant when evaluated in a model containing body weight and temperature (Table 4). However, in the model that included THI to predict DWI [root mean square error (RMSE) = 2.83 L/d], DMI and not body weight was retained in the model (Table 5). The correlation between body weight and DMI was 0.371 (*P* < 0.001) and this collinearity may contribute to the inclusion or exclusion of DMI in models. The inclusion of DMI or body weight in a model is influenced by the experiments that report this variable; hence it is not just the importance of a variable to the model but also the distribution of variables included in experiments that influences final model selection. Loneragan et al. (2001) and Sexson et al. (2012) also identified univariable associations for DWI with DMI. However, DMI did not enter the multivariable models for Sexson et al. (2012), while Loneragan et al. (2001) included both DMI and body weight in a model that predicted DWI. Loneragan et al. (2001) also included mean daily temperature, barometric pressure, water sulfate, wind speed, and humidity. Hicks et al. (1988) also included DMI in a multivariable model that included precipitation, maximum daily temperature, and dietary salt content. Meyer et al. (2006) included DMI in a model of intake for Holstein bulls that also included average ambient temperature, the roughage percentage of the diet, dry matter content of the roughage, and body weight. Machado et al. (2021) developed DWI models for *Brangus heifers* that included both DMI and body weight.

**Table 3:** Univariable estimates for factors influencing drinking water intake including the random effects of study and experiment.

Variable	No. of studies	No. of experiments	Constant	Coefficient	*P*-value	Heterogeneity study	Heterogeneity experiment	Overall heterogeneity
**Temperature, ^o^C**	21	82	17.9	0.696	<0.001	54.5	45.1	99.6
**Relative humidity, %**	15	58	35.2	−0.009	0.967	49.2	50.6	99.9
**Temperature humidity index**	18	68	1.25	0.497	<0.001	54.1	45.6	99.7
**Wind speed, m/s**	10	37	27.9	0.772	0.294	47.9	49.1	97.0
**Heat load index**	9	32	−20.4	0.768	<0.001	85.0	9.53	94.6
**Weight, kg**	32	123	17.2	0.042	<0.001	63.1	36.5	99.6
**Average daily gain, kg/d**	21	74	37.0	−1.958	0.536	92.2	1.16	93.7
**Dry matter intake, kg**	30	114	25.9	0.873	0.013	55.0	44.6	99.6
**Diet dry matter, %**	29	111	0.232	0.437	<0.001	44.3	55.3	99.6
**Water sulfur, g/d**	7	28	34.8	−0.027	0.237	74.4	10.1	84.5
**Shade (Yes or no [no includes not stated])**	33	127	33.2	1.891	0.467	58.2	41.4	99.6
**Sex**	34	129	33.3			47.5	52.5	100
**Bull (reference group)**		13		(ref)				
**Heifer**		22		−3.670	0.645			
**Mixed**		14		−2.588	0.738			
**Steer**		82		−0.559	0.937			

**Table 4:** Final model to predict drinking water intake for feedlot cattle when average daily temperature is greater than 12 °C for 20 studies and 75 experiments.^1^

Variable	Coefficient	Standard error	*P*-value	Random effects	Heterogeneity *I*^2^	Pseudo *R*^2^
**Temperature, ^o^C**	1.32	0.21	<0.001			
**Body weight, kg**	0.043	0.012	<0.001			
**Constant**	−14.9	7.31	0.042			
**Study**				6.732	54.6	0.428
**Experiment**				6.099	44.8	0.295
**Overall**					99.5	0.375

1 The drinking water intake can be estimated using the constants below. The RMSE was 2.40 L/d.

**Table 5:** Final model to predict drinking water intake for feedlot cattle when using temperature humidity index and dry matter intake for 14 studies and 57 experiments.^1^

Variable	Coefficient	Standard error	*P*-value	Random effects	Heterogeneity *I*^2^	Pseudo *R*^2^
**Temperature humidity index**	0.609	0.097	<0.001			
**Dry matter intake, kg/d**	1.39	0.420	0.001			
**Constant**	−19.6	9.31	0.035			
**Study**				8.943	58.9	0.353
**Experiment**				7.450	40.8	0.455
**Overall**					99.9	0.399

1 The drinking water intake can be estimated using the constants below. The RMSE was 2.83 L/d.

The univariable estimates provided in Table 3 identified factors that were highly significant predictors of DWI (*P* < 0.001) including temperature, THI, body weight, HLI, dietary dry matter percentage, and DMI (*P* = 0.013). The pseudo-*R*^2^ estimates for the overall models suggest good fit for all models except relative humidity; however, the pseudo-*R*^2^ include the effects of study and experiment, not just the variable under evaluation. The RMSE for temperature, wind speed, body weight and water sulfur were all low (≤2.8 L/d). All models had considerable heterogeneity, with most models exceeding 95% and only sulfur being less than this with an overall heterogeneity of 84.5%. The high heterogeneities are consistent with the differences in THI and temperature across the wide range of study locations, study designs, level at which measures were taken (pen or individual), and methods of measurement.

**Figure 3:**
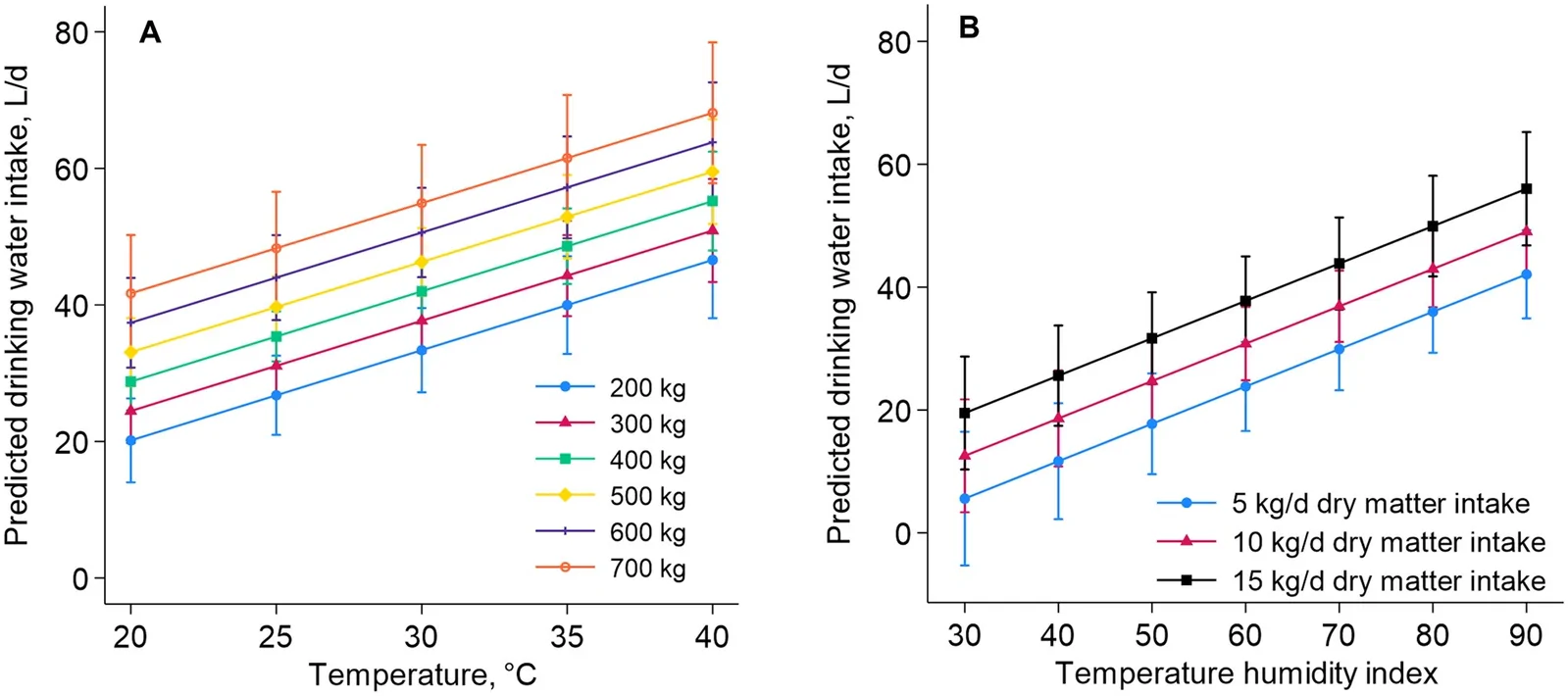
A) The relationship between water intake (L/d), temperature (°C), and body weight (kg) based on least square means from the meta-regression model with 95% confidence intervals. There was no significant interaction between temperature and body weight hence the linear estimates for body weight with change in temperature. B) The relationship between water intake (L/d), temperature humidity index, and dry matter intake (kg/d) based on least square means from the meta-regression model with 95% confidence intervals. The was no significant interaction between temperature humidity index and dry matter intake hence the linear estimates for dry matter intake with change in temperature humidity index.

The considerable heterogeneity in evaluation of the factors influencing DWI suggest that the external validity of findings from this study will be high as such a wide range of study conditions that influence DWI was present. The estimations of DWI for temperatures over 12°C and body weight in the model developed from the study data (Table 4; Figure 3A; RMSE = 2.40 L/d) had a Lin’s concordance correlation coefficient *ρ*_c_ (Lin 1989) of 0.462, a Pearson’s correlation of 0.936, and the slope for the major axis was 1.62 with an intercept of −4.13 L/d with the estimates of DWI in the NRC (2016). Standardized residuals from the model (Table 4) did not deviate by 1 SD from the reported DWI. Removal of the large study of Sexson et al. (2012) or single studies with larger residuals did not confound coefficients nor significance of results. Similarly, removal of Latin square studies did not confound coefficients nor significance of results. Figure 3B shows the relationship of DWI with increased THI and Table 5 provides estimates for the effects of THI and DMI on DWI.

### Model comparisons

All variables with a *P* value of <0.2 were tested using Spearman’s rank correlations (data not shown); temperature and THI had r values of 0.977. Arias and Mader (2011) also identified collinearity for THI and average temperature. The THI was dropped for a model that examined effects of average temperature, and collinearity for the remaining variables had a combined variance inflation factor of 4.03, indicating moderate correlation. The temperature (Table 4; RMSE = 2.40 L/d) and THI (Table 5; RMSE = 2.83 L/d) models were tested for fit based Akaike information criterion and Bayesian information criterion. Considering the greater number of studies based on temperature and the lower RMSE for that model rather than THI the favored model was that based on temperature and body weight (Table 4). This decision is supported by the similarity in prediction (*R*^2^ = 0.57) for both THI and average temperature identified in the large study of Arias and Mader (2011). Evaluation of standardized residuals indicated that the fit of models was poor for temperature <12 °C and this is consistent with a lack of stimulus for increased DWI with temperatures below 12°C and the threshold of thermal stress effect discussed by Wijffels et al. (2024). There was no evident deviation from linearity or deviation in residuals for studies greater than 12°C in our data (data not shown) and all fell within 1 SD of reported DWI. Further, DWI has been modelled to account for seasonal effects (Arias and Mader 2011) reflecting a threshold effect identified in that study for increased DWI at a THI threshold of 67.2. Sensitivity analysis for our model found that it was robust to removal of individual large studies or studies with large residuals and removal of Latin squares as neither coefficients for temperature and body weight were confounded. Given the significance and importance of body weight in many of the studies, we elected to use the model that included studies with possible pseudo-replication, where insufficient detail was provided to determine whether pseudo-replication had occurred.

**Figure 4:**
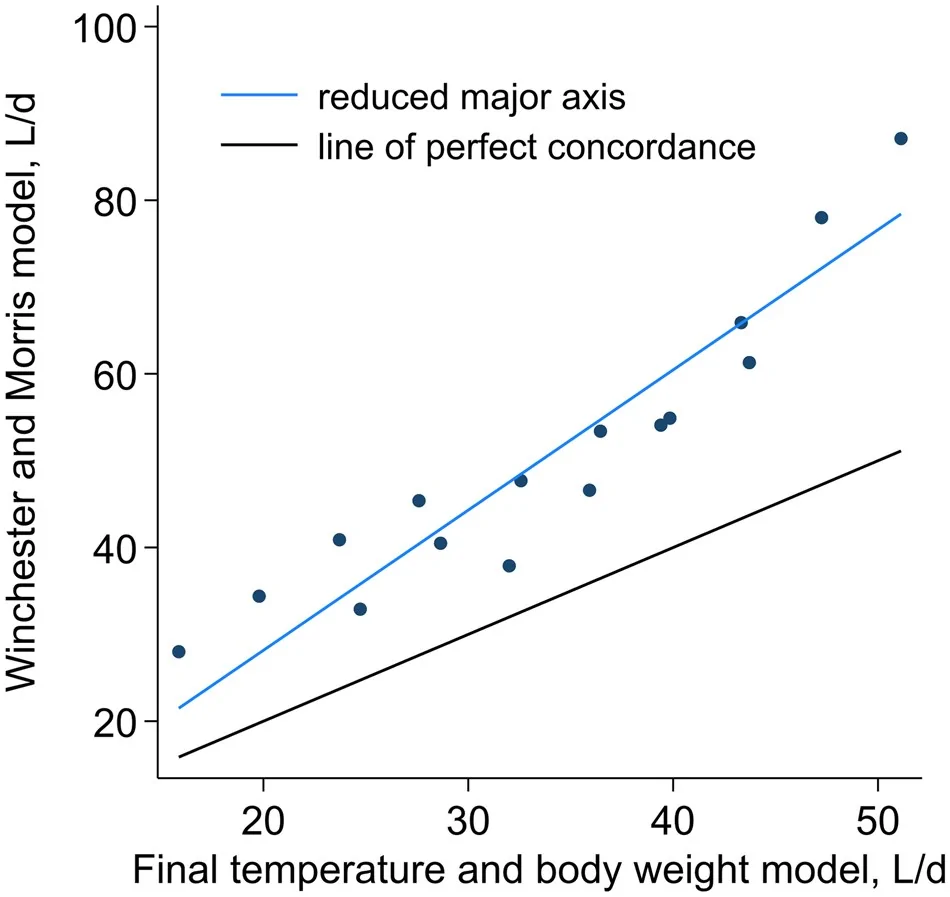
The concordance between drinking water intake (L/d) estimated from Winchester and Morris (1956) in NRC (2016) and predicted estimates from our final temperature (^o^C) and body weight (kg) model in Table 4.

Predicted estimates of DWI derived from the final model with temperature and body weight (Figure 4) are much lower than those in the NRC (2016) despite the correlation between values being high. Bland and Altman’s (1986) limits of agreement showed that the mean difference between predicted estimates was 16.7 L/d (SD = 7.70) with 95% limits of agreement of 1.60–31.8 L/d. Hicks et al. (1988) estimated that DWI modeled using Winchester and Morris (1956) would be 10–15% greater than observed in their study. The Hicks et al. (1988) model included maximum daily temperature, DMI, precipitation, and salt percentage in the diet as a percentage of dry matter and had an *R*^2^ of 0.74. Arias and Mader (2011) found an *R*^2^ of 0.65 across seasons for two models developed in a large multisite study in finishing cattle which predicted DWI from 1) DMI, solar radiation, and minimum temperature; or 2) DMI, solar radiation, and THI. The higher *R*^2^ for the Hicks et al. (1988) model than the models in our study probably reflect the more consistent data obtained from a single site study. Similarly, the much larger data set from Arias and Mader (2011) was from a single site but included differing years and underlying experimental design. Machado et al. (2021) developed a model for DWI that included DMI, relative humidity, maximum temperature, THI, and body weight, with an adjusted *R*^2^ of 0.42 and RMSE of 6.65. These models (Table 6; Hicks et al. 1988; Arias and Mader 2011; Machado et al. 2021) and others from single sites will have strong internal validity but be less generalizable than the models from this study.

**Table 6:** Comparisons among studies and reports presenting models of drinking water intake.

Study	Water intake (L/d)	Body weight (kg)	Temperature (^o^C)	Dry matter intake (kg/d)	Water intake (L/d)/kg dry matter intake
**NRC (2016) for temperature >12 ^o^C**	50.6	408	23.6	9.7	5.2
**Current temperature and body weight**	33.3	392	25.1	8.5	3.9
**Current temperature humidity index and dry matter intake**	32.1	417	21.6	9.1	3.5
**Hicks et al. (1988) (0% dietary salt intake)**	38.5	411	24.2	9.0	4.3
**Hicks et al. (1988) (0.25% dietary salt intake)**	34.0	404	24.2	8.4	4.0
**Hicks et al. (1988) (0.5% dietary salt intake)**	35.3	408	24.2	8.5	4.2
**Arias and Mader (2011)**	32.4	∼450	21.4	9.6	3.4
**Sexson et al. (2012)**	37.1	468	22.0	9.9	3.7
**Machado et al. (2021)**	12.7	229	19.5	5.1	2.5

### Effects of sulfur

**Figure 5:**
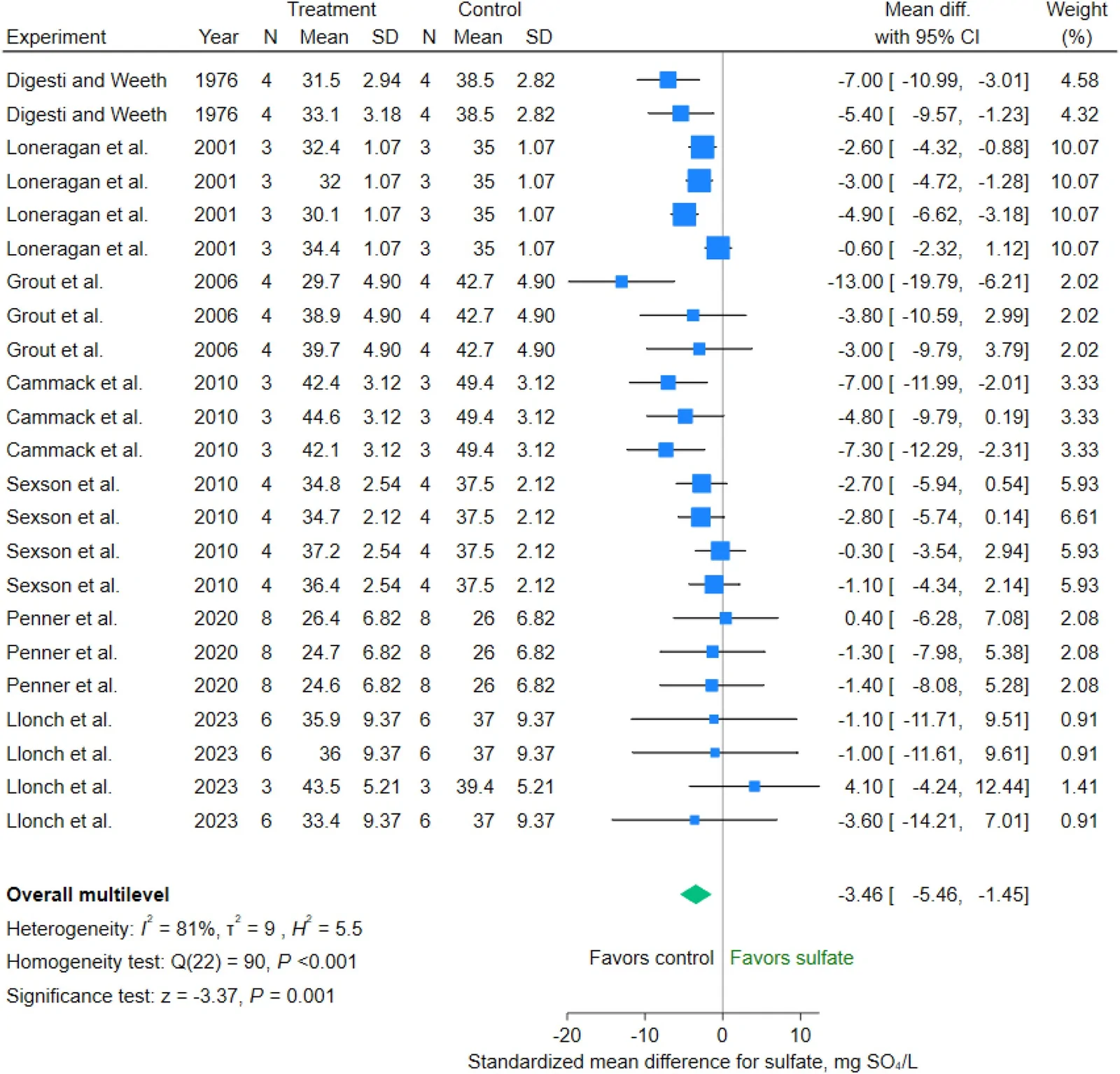
Forest plot of the standardized mean difference (SMD) and 95% CI of the effect on drinking water intake (L/d) for feedlot cattle treated with water or feed containing sulfate. The solid vertical line represents a mean difference of zero or no effect. Points to the left of the line represent a reduction in final drinking water intake (DWI), while points to the right of the line indicate an increase. Each square around the point effect represents the mean effect size for that comparison at the univariate level and reflects the relative weighting of the comparison to the overall effect size estimate. The larger the box, the greater the comparison contribution to the overall SMD estimate. The weights that each comparison contributed at the univariate level are in the right-hand column. The upper and lower limit of the line connected to the square represents the upper and lower 95% CI for the SMD. The overall pooled SMD and 95% CI pooled using the DerSimonian and Laird (1986) and multilevel meta-analytical random effects models (Goldstein et al. 2000) are indicated by the univariate and multilevel respective diamonds at the bottom. The estimate of the overall effect size θ for the multilevel model is reported as the significance test and indicates a significant effect in reducing DWI (*P *< 0.01). The heterogeneity measure *I*^2^ is a measure of residual variation among experiments included in the meta-analysis. The final DWI was considerably heterogenous as indicated by the overall *I*^2^ of 81.0% estimated from the multilevel model. The *τ*^2^ is the true variance between the total of studies and experiments and is high. The *H*^2^ also provides an estimate of heterogeneity. The *H* estimated from the multilevel model is interpreted as the ratio of the SD of the estimated overall effect size from a random effects meta-analysis compared to the SD from a fixed effects meta-analysis, where a *H*^2^ = 1 is homogenous. In this case, the *H*^2^ is heterogenous.

**Figure 6:**
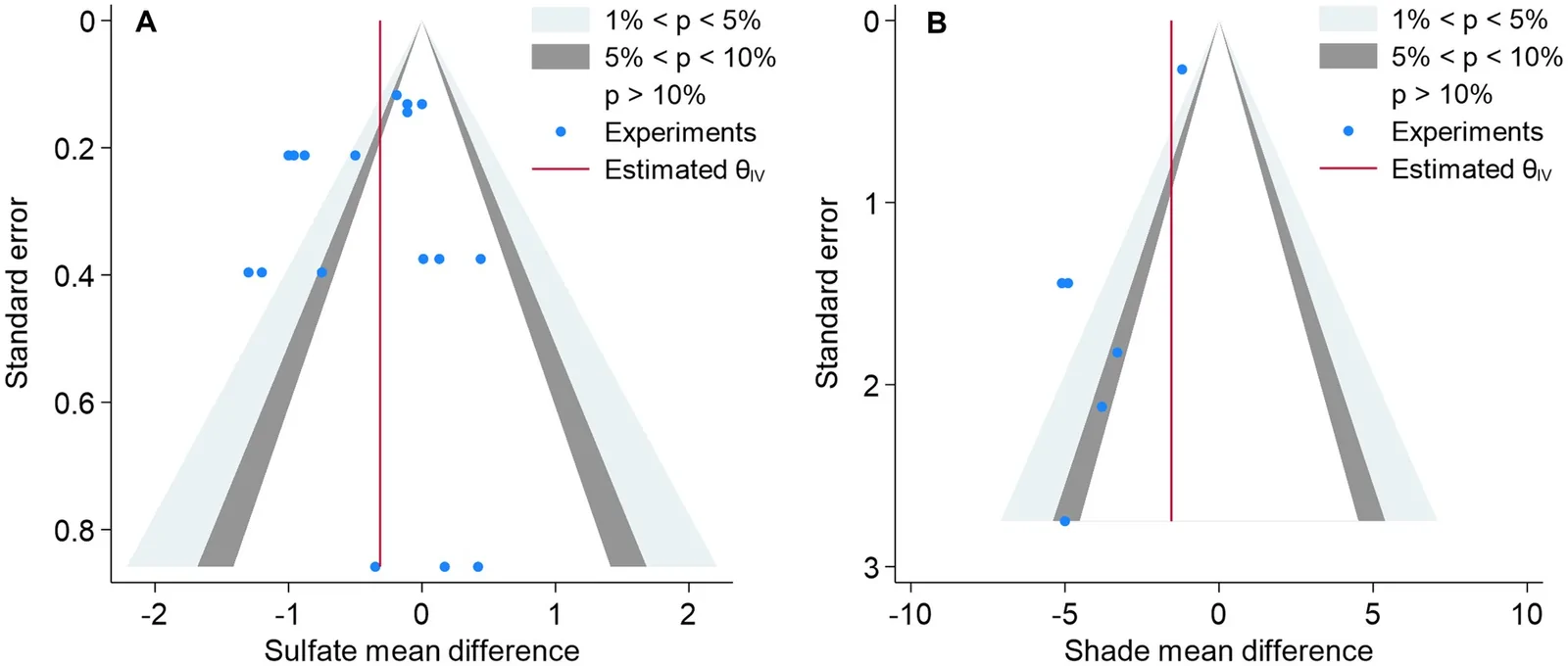
Contour-enhanced funnel plot for drinking water intake for cattle A) treated with water or feed containing sulfate or B) provided shade. The dots are the effect estimates of the responses of interest and their respective SEM from the experiments. Levels of significance for experiments and within the solid lines are 0.01, 0.05, and 0.10.

There were seven studies and 23 experiments that investigated the effect of sulfur content of water on DWI. Figure 5 shows that water sulfur content (mg SO_4_/L) reduces DWI. When the difference in sulfur content (mg SO_4_/L) between treatment and control groups was accounted for in a multilevel model, there was a decrease in DWI by 0.2 liter for every 100 mg/L increase in water sulfate (SO_4_) (Table 7). This effect was not significant if the one experiment of Llonch et al. (2023) with the greatest difference in sulfur (mg SO_4_/L) between treatment and control was excluded but the univariable *leaveoneout* analysis did not support the exclusion of the experiment. Consequently, we report the effect from the multilevel model including all experiments. There was no evidence of publication bias in the funnel plot (Figure 6A). The effect of sulfur content in the water was not significant on ADG. However, sulfur content in the water was significant on univariable analysis (*P* < 0.001), but not multilevel analysis for DMI (Table 7) indicating that study had a very substantial influence on the effects of sulfur content in the water on DMI.

**Table 7:** Effect of difference in sulfur content in drinking water or feed (mg SO_4_/L) between treatment and control on drinking water intake, average daily gain, and dry matter intake.

Variable	No. of studies	No. of experiments	Constant	Coefficient	*P*-value	Heterogeneity study	Heterogeneity experiment	Overall heterogeneity
**Water intake, L/d**	7	23	−1.80	−0.002	0.002	59.0	18.9	78.0
**Average daily gain, kg/d**	6	17	−5.00e^−3^	−3.00e^−5^	0.860	0	0	0
**Dry matter intake, kg/d**	5	17	−0.43	0.0002	0.684	45.5	0	45.5

**Figure 7:**
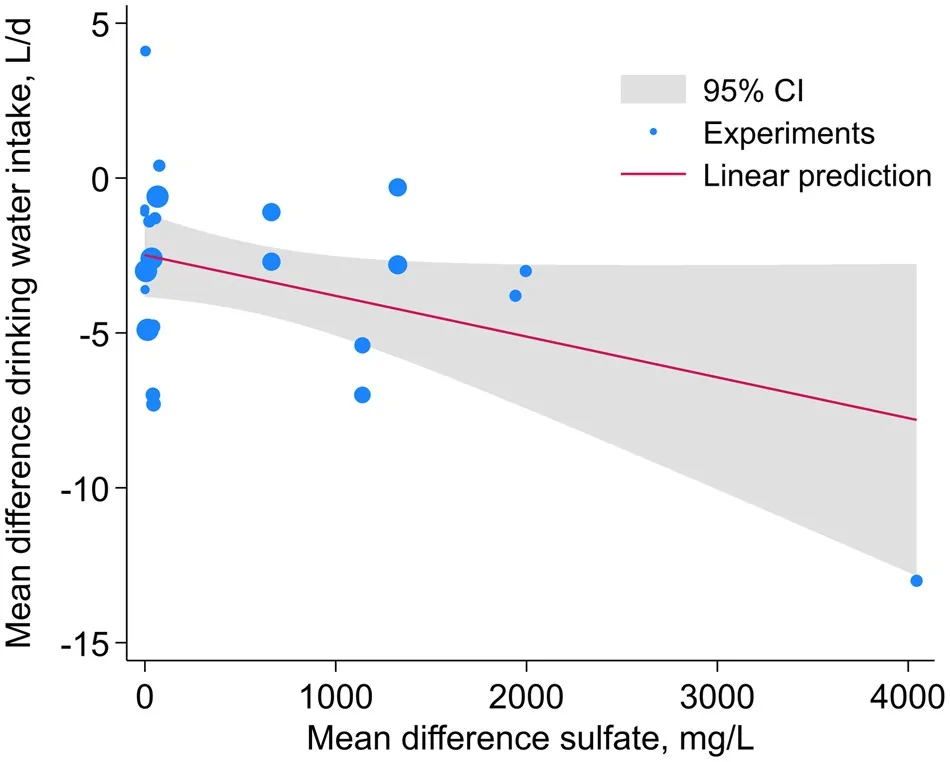
Bubble plot of drinking water intake (L/d) for cattle treated with water or feed containing sulfate. The circular markers are the weighted effect estimates from a univariable estimate of the responses in the 23 experiments. The red line indicates the linear prediction for DWI as sulfate in the water increases (*P* = 0.07).

The variability in response to sulfur content in the water in different studies has been noted (Wagner and Engle 2021) and the number of studies and experiments is limited with only seven studies meeting the criteria for inclusion in this study. However, these replicated studies represent those specifically designed to address sulfur content in water in feedlots and provide greater study power and can be more readily generalized than a single study. Concentrations of sulfate were not substantially high with only Llonch et al. (2023) providing 4,000 mg SO_4_/L and other studies being <3,000 mg SO_4_/L. The NRC (2016) recommendation is that calves have water with <500 mg/L of sulfate and that adult cattle have <1,000 mg/L of sulfate but that form of sulfate is important. The regressions on water intake in Figure 7 and the univariable effect on DMI support the current recommendations from NRC (2016) that water sulfate content is a potentially important risk for feedlot cattle.

### Effect of shade

**Figure 8:**
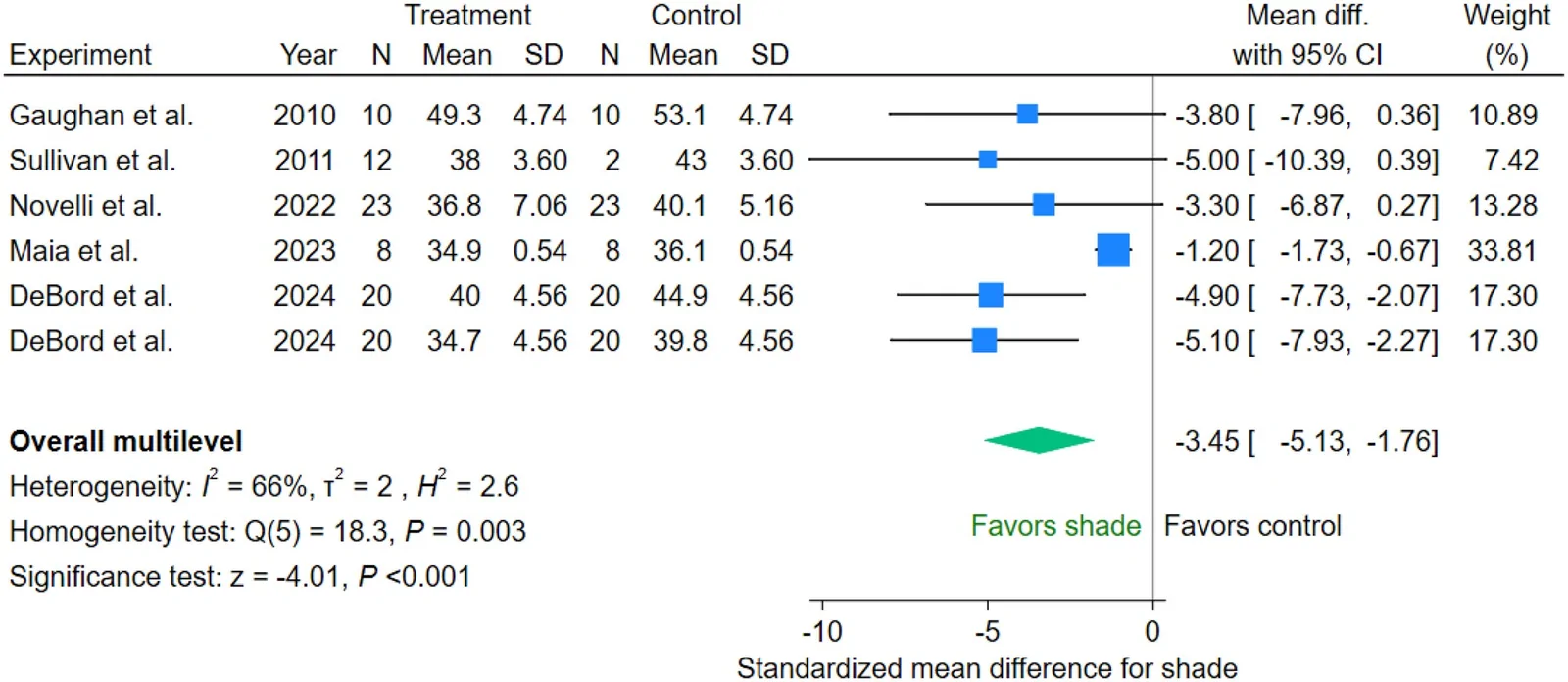
Forest plot of the standardized mean difference (SMD) and 95% CI of the effect on drinking water intake (DWI; L/d) for proving shade for feedlot cattle. The solid vertical line represents a mean difference of zero or no effect. Points to the left of the line represent a reduction in final DWI, while points to the right of the line indicate an increase. Each square around the point effect represents the mean effect size for that comparison at the univariate level and reflects the relative weighting of the comparison to the overall effect size estimate. The larger the box, the greater the comparison contribution to the overall SMD estimate. The weights that each comparison contributed at the univariate level are in the right-hand column. The upper and lower limit of the line connected to the square represents the upper and lower 95% CI for the SMD. The overall pooled SMD and 95% CI pooled using the DerSimonian and Laird (1986) and multilevel meta-analytical random effects models (Goldstein et al. 2000) are indicated by the univariate and multilevel respective diamonds at the bottom. The estimate of the overall effect size *θ* for the multilevel model is reported as the significance test and indicates a significant effect in reducing DWI (*P *< 0.01). The heterogeneity measure, *I*^2^ is a measure of residual variation among experiments included in the meta-analysis. The final DWI was considerably heterogeneous as indicated by the overall *I*^2^ of 66.0% estimated from the multilevel model. The *τ*^2^ is the true variance between the total of studies and experiments and is moderately high. The *H^2^* also provides an estimate of heterogeneity. The *H* estimated from the multilevel model is interpreted as the ratio of the SD of the estimated overall effect size from a random effects meta-analysis compared to the SD from a fixed effects meta-analysis, where a *H^2^* = 1 is homogenous. In this case, the *H*^2^ is heterogenous.

There were 5 studies and 6 experiments that specifically investigated the effect of shade on DWI, whereas shade use was noted in 33 studies and 127 experiments; 28 of the 33 studies did not have this as a specific treatment effect. The data from Sullivan et al. (2011) were re-organized to allow a single evaluation reflecting the presentation of their results. The evaluation of shade in the five studies and six experiments that specifically investigated its effects was more precise than using the reported exposures from a large number of studies. Figure 8 and Table 8 show that shade reduces DWI by 3.4 L/d. There was no evidence of publication bias in the funnel plot (Figure 6B). Table 8 shows no significant effect of shade on ADG or DMI with the limited number of studies and experiments.

**Table 8:** Effects of shade for treatment and control on drinking water intake, average daily gain, and dry matter intake.

Variable	No. of studies	No. of experiments	Coefficient	*P*-value	Heterogeneity study	Heterogeneity experiment	Overall heterogeneity
**Water intake, L/d**	5	6	−3.45	<0.001	66.2	0.0	72.7
**Average daily gain, kg/d**	5	6	0.11	0.362	0	0	0
**Dry matter intake, kg/d**	5	6	0.07	0.723	0	0	0

## Conclusions

In conclusion, our objectives were met with successful evaluation of DWI of cattle in feedlots and our hypothesis was met with DWI reflecting body weight and environment. While many of the 35 studies with up to 131 experimental comparisons were observational others evaluated a variety of interventions including water quality and shade and studies were conducted in backgrounding, induction, and finishing stages of the feedlot. The study base was both substantial and highly varied suggesting that the results should be strongly applicable to feedlots. Two final models to predict water intake for feedlot cattle were developed 1) with temperature and body weight both independently predicting DWI when average daily temperature is greater than 12°C based on 20 studies and 75 experiments (RMSE = 2.40 L/d). 2) The second model had THI and dry matter intake independently predicting DWI based on 14 studies and 57 experiments (RMSE = 2.83 L/d). Estimates of DWI derived using both models are much lower than those in the NRC (2016). The favored model was that with temperature and body weight. The substantial residual heterogeneity; however, suggests that other factors, such as water quality, breed, sex, shade, and duration of heat challenge, that were not significant in the final model for DWI influenced water intake and should be considered in water allocation to cattle, experimental, and facility design. Drinking water intake is reduced by 3.4 L/d when shade is provided. Drinking water intake decreased by 0.2 liter for every 100 mg/L increase in water sulfate (SO_4_). We provide updated robust estimates of changes in DWI for feedlot cattle with increases in environmental temperature, THI, body weight, and feed intake that improve physiological understandings of DWI to guide industry in water allocation and improve cattle welfare.

## Supplementary Material

skag263_Supplementary_Data

## Abbreviations

ADG: average daily gain
AHLU: accumulated heat load units
CCI: cattle comfort index
D&L: DerSimonian and Laird (1986) random effects models
DM: dry matter
DMI: dry matter intake
DWI: drinking water intake
HLI: heat load index
RMSE: root mean square error
SMD: standardized mean difference
THI: temperature humidity index
