# Supplementary material for: Drinking water intake in feedlot cattle: a meta-analysis
Source: J Anim Sci. 2026 Sep 1;104:skag263. doi: 10.1093/jas/skag263 (PMC13592467; doi:10.1093/jas/skag263)
Supplement: Supplementary file 1 [file skag263_supplementary_data.docx]

**Table S1** Author and reason for exclusion

| **Authors** | **Reason for exclusion** |
| --- | --- |
| Ahlberg (2018) | Companion thesis |
| Ahlberg et al. (2018) | Companion research article |
| Beatty et al. (2006) | No suitable data |
| Broadway et al. (2020) | Data not able to be extracted |
| Davis et al. (2003) | Companion research article |
| Harper et al. (1997) | Off topic research article – coal mine pit water |
| Moehlenpah et al. (2021) | Off topic research article – lactating cattle |

**References**

Ahlberg, C. M. 2018. Characterization of water intake in beef cattle: Test length guidelines, water intake prediction, and genetic parameters, Kansas State University PhD Dissertation.

Ahlberg, C. M., K. Allwardt, A. Broocks, K. Bruno, L. McPhillips, A. Taylor et al.,. 2018. Test duration for water intake, ADG, and DMI in beef cattle. J. Anim. Sci. 96:3043-3054. doi:<https://doi.org/10.1093/jas/sky209>

Beatty, D. T., A. Barnes, E. Taylor, D. Pethick, M. McCarthy, and S. K. Maloney. 2006. Physiological responses of *bos taurus* and *Bos indicus* cattle to prolonged, continuous heat and humidity. J. Anim. Sci. 84:972-985. doi:<https://doi.org/10.2527/2006.844972x>

Broadway, P. R., J. A. Carroll, N. C. Burdick Sanchez, M. D. Cravey, and J. R. Corley. 2020. Some negative effects of heat stress in feedlot heifers may be mitigated via yeast probiotic supplementation. Front. Vet. Sci. 6:515. doi:<https://doi.org/10.3389/fvets.2019.00515>

Davis, M. S., T. L. Mader, S. M. Holt, and A. M. Parkhurst. 2003. Strategies to reduce feedlot cattle heat stress: Effects on tympanic temperature. J. Anim. Sci. 81:649-661. doi:<https://doi.org/10.2527/2003.813649x>

Harper, G. S., T. J. King, B. D. Hill, C. M. L. Harper, and R. A. Hunter. 1997. Effect of coal mine pit water on the productivity of cattle .2. Effect of increasing concentrations of pit water on feed intake and health. Aust. J. Agric. Res. 48:155-164. doi:<https://doi.org/10.1071/a96067>

Moehlenpah, A. N., L. P. S. Ribeiro, R. Puchala, A. L. Goetsch, P. Beck, A. Pezeshki et al.,. 2021. Water and forage intake, diet digestibility, and blood parameters of beef cows and heifers consuming water with varying concentrations of total dissolved salts. J. Anim. Sci. 99:1-10. doi:<https://doi.org/10.1093/jas/skab282>
